# Integration of mate pair sequences to improve shotgun assemblies of flow-sorted chromosome arms of hexaploid wheat

**DOI:** 10.1186/1471-2164-14-222

**Published:** 2013-04-04

**Authors:** Tatiana Belova, Bujie Zhan, Jonathan Wright, Mario Caccamo, Torben Asp, Hana Šimková, Matthew Kent, Christian Bendixen, Frank Panitz, Sigbjørn Lien, Jaroslav Doležel, Odd-Arne Olsen, Simen R Sandve

**Affiliations:** 1 Department of Plant and Environmental Sciences, University of Life Sciences, Ås, Norway; 2 The Genome Analysis Centre (TGAC), Norwich Research Park, Norwich NR4 7UH, UK; 3 Department of Molecular Biology and Genetics, Aarhus University, Forsøgsvej 1, 4200, Slagelse, Denmark; 4 Centre of the Region Haná, Institute of Experimental Botany, 77200, Olomouc, Czech Republic; 5 Centre for Integrative Genetics (CIGENE) and Department of Animal and Aquacultural Sciences, Norwegian University of Life Sciences, Ås N-1432, Norway; 6 Department of Genetics and Biotechnology, Faculty of Agricultural Sciences, Aarhus University, Tjele 8830, Denmark

**Keywords:** Wheat, Assembly, Scaffold, Mate-pair, MDA, Improvement

## Abstract

**Background:**

The assembly of the bread wheat genome sequence is challenging due to allohexaploidy and extreme repeat content (>80%). Isolation of single chromosome arms by flow sorting can be used to overcome the polyploidy problem, but the repeat content cause extreme assembly fragmentation even at a single chromosome level. Long jump paired sequencing data (mate pairs) can help reduce assembly fragmentation by joining multiple contigs into single scaffolds. The aim of this work was to assess how mate pair data generated from multiple displacement amplified DNA of flow-sorted chromosomes affect assembly fragmentation of shotgun assemblies of the wheat chromosomes.

**Results:**

Three mate pair (MP) libraries (2 Kb, 3 Kb, and 5 Kb) were sequenced to a total coverage of 89x and 64x for the short and long arm of chromosome 7B, respectively. Scaffolding using SSPACE improved the 7B assembly contiguity and decreased gene space fragmentation, but the degree of improvement was greatly affected by scaffolding stringency applied. At the lowest stringency the assembly N50 increased by ~7 fold, while at the highest stringency N50 was only increased by ~1.5 fold. Furthermore, a strong positive correlation between estimated scaffold reliability and scaffold assembly stringency was observed. A 7BS scaffold assembly with reduced MP coverage proved that assembly contiguity was affected only to a small degree down to ~50% of the original coverage.

**Conclusion:**

The effect of MP data integration into pair end shotgun assemblies of wheat chromosome was moderate; possibly due to poor contig assembly contiguity, the extreme repeat content of wheat, and the use of amplified chromosomal DNA for MP library construction.

## Background

Bread wheat is one of the most important food crops worldwide. However, present wheat production is far from the expected increased global demand in the near future [1,2]. Development of better yielding varieties with improved adaptation to the new climatic challenges is therefore important for global food security. A ‘tool’ with a great potential to revolutionize wheat breeding and production is a publicly available reference genome sequence. Genome sequences enable cost-effective identification of genomic variation which subsequently can be used to improve agricultural traits of interest through marker-assisted selection (MAS) and genomic selection programs [3]. A rapidly increasing number of genomes from important food crops are becoming available. In 2011 potato and cacao [4,5], in 2010 soybean [6], and in 2009 maize, sorghum and cucumber genomes were published [7-9]. However, even though wheat is one of the top five food commodities in the world, a wheat genome sequence is not yet available.

The main reason why the wheat genome sequencing is lagging behind is related to technical challenges due to large size (17Gb) and the complexity of the hexaploid wheat genome. Bread wheat is allohexaploid and carries three distinct, but closely related homoelogous genomes (2*n* = 6*x* = 42, AABBDD) [10,11]. A distinction between homoeolog sequences in post sequencing processing of genomic sequence data is essentially impossible. Fortunately, the hexaploid wheat genome can be dissected to small parts by flow cytometric sorting of single chromosomes and chromosome arms [12,13]. This technological breakthrough has enabled production of wheat chromosome specific BAC-libraries [14] and facilitated construction of physical maps of hexaploid wheat chromosomes [15]. For some genomic applications, such as shotgun sequencing, large amount of DNA are required. In order to obtain sufficient DNA to sequence purified chromosome arms, millions of chromosomes must be sorted, a process, which is highly labor intensive [16]. Including an amplification step of flow-sorted DNA can significantly reduce the labor and consequently the cost of acquiring chromosome specific DNA for sequencing. Multiple displacement amplification (MDA) is the most common method for genome amplification for sequencing purposes as MDA generate relatively long amplification products (majority between 5-20 kb) [17]. However, MDA is known to give rise to chimeras, which can bring down the utility of the amplified DNA [18].

Shotgun sequencing of MDA DNA from flow-sorted chromosome arms, especially in combination with genetic maps and synteny information, has proven to be a highly cost effective way of gene discovery and construction of syntenic chromosome assemblies [19-21]. Unfortunately, the fragmentation level of the shotgun assemblies has been very high, which limits the information value of the assemblies. *De novo* assemblies of 7DS and 7BS using Illumina paired-end (PE) sequences with a chromosome arm coverage of 30-34×, resulted roughly in 600,000-1,000,000 contigs per chromosome arm, an N50 of ~500-1200 bp, and maximum contig sizes of just over 30,000 bp [21,22]. Consequently, many contigs do not contain complete gene sequences, and the relative order of genes can only be identified for a small subset of genes found on contigs containing multiple genes (i.e. multigene contigs).

High levels of DNA sequence assembly fragmentation is closely associated with the repeat content of the genome [23], and the wheat genome is extreme with respect to repeat content, having more than 80% repetitive DNA [24]. One way of reducing assembly fragmentation is to include additional sequencing libraries with large insert sizes, referred to as mate pair (MP) libraries [23]. MP reads can vary in insert sizes between 1-20 kb and the idea of these ‘long jump’ paired sequences is to span repetitive regions that cause assembly fragmentation, and thereby link multiple contigs into longer scaffolds. This will improve the information value of an assembly by (1) improving the assembly contiguity (2) increasing the proportion of full length genes contained in single sequences (i.e. link exons from different contigs), and (3) increase the number of linearly ordered genes.

A number of recent publications describe the effect of MP data on assemblies of plant genomes [4,9,25]. One example is the potato genome assembly, which had on average an N50 increase of 37 Kb for every 1 Kb increase in MP insert size [25]. Although the potato genome (1C = 865 Mbp) has a relatively high repeat content (total repeat content ≈ 62%, TE-derived repeats ≈ 32%), it does not compare to the hexaploid wheat genome (1C = 17,000 Mbp) that has >80% of TE-derived repetitive DNA [24]. It is thus not clear to what extent MP data may improve shotgun assemblies of genomes with extreme repeat content such as wheat. Additionally, the utility of MP data from MDA DNA from flow-sorted chromosomes is unknown. The aim of this paper is therefore to study the effects of MP from MDA DNA on assembly contiguousness and gene content in shotgun assemblies of a flow-sorted hexaploid wheat chromosome.

## Methods

### Preparation of DNA from chromosome arms 7BS and 7BL

A double ditelosomic line of wheat *Triticum aestivum* L. cv. Chinese Spring carrying both arms of chromosome 7B as telosomes (2n = 40 + 2t7BS + 2t7BL) was used to purify the 7BS and 7BL arms. The seeds were provided by Dr. Bikram Gill (Kansas State University, Manhattan, USA). The chromosome arms were purified by flow cytometry. 68,000 and 45,000 of 7BS and 7BL arms, respectively, corresponding to 50 ng of DNA, were isolated in several batches. In order to estimate contamination with other chromosomes, 1000 chromosomes were sorted onto a microscope slide and used for fluorescence *in situ* hybridization (FISH) with probes for *Afa* family and telomeric repeats. Batches with the highest purity of the sorted fraction (93 and 88% for 7BS and 7BL, respectively) were used for further processing. DNA was purified and subsequently amplified using Illustra GenomiPhi V2 DNA Amplification Kit (GE Healthcare, Chalfont St. Giles, United Kingdom) as previously described [17]. Three independent amplifications were performed for each arm to reduce amplification bias. Totally, 15.9 and 14 micrograms were prepared for 7BS and 7BL, respectively.

### Sequencing library construction

PE libraries with a mean insert size of ~350 bp (Illumina protocol) and 2 Kb MP libraries (in-house modified Roche MP protocol) were constructed and sequenced at Fasteris SA (Geneva, Switzerland). The PE reads were 100 bp, while the 2 Kb MP reads were 45 bp. 3 Kb and 5 Kb MP libraries were prepared according to “Mate Pair Library v2 Sample Preparation Guide” [26] at Aarhus University (Denmark). The read length of the 3 and 5 Kb MP libraries were trimmed to 35 bp. All MP libraries were sequenced using HiSeq2000 technology (Illumina) according to manufacturer’s recommendations.

### Contig assembly

Contigs were assembled with PE reads using ABySS [27] which is based on a *de Bruijn* graph approach. This method collects the information generated from fix-length words of *k –mers* shared by overlapping reads [28]. Initially, multiple assemblies were generated using different values of *k* and assessed using assembly quality statistics such as N50, maximum contig length, number of contigs in the assembly and the total amount of bases in the assembly. A *k-mer* length of 71 was chosen as the optimal value. A seed value of 150 was used (s parameter) and a minimum of 10 pairs were required to join contigs (n parameter). After assembly, contigs shorter than 200 bp were removed to generate a filtered dataset for scaffolding.

### Scaffold assembly

To accurately determine the mean insert size and insert size variation of each MP library, we mapped all mate pair reads back to the 7B contigs using BWA v0.6.0 [29] with the parameters BWA aln –t 10 -q 10. Based on the BWA results we identified the number of MP reads aligning to contigs, the proportion of MP read pairs mapping to the same or different contigs, and the orientation of the MP reads that mapped to the same contig. We also assessed if the genomic origin of MP reads were biased towards different fractions of the genome (i.e. repeat or conserved fraction). This was done by mapping reads to an in house repeat content database (TREP10 and the repeats identified in Choulet et al. 2011) and the NCBI nr database.

We initially tested three software packages for scaffolding of pre-assembled contigs: ABySS, SOAPdenovo and SSPACE. Unfortunately, we were not able to scaffold contigs using ABySS due to the large proportion of MP reads that mapped in forward-forward direction (see results and discussion for more details). SOAP and SSPACE both produced scaffolds, but as the N50 and gene space assembly statistics of SSPACE assemblies exceeded SOAPdenovo at all parameters tested, we chose to use SSPACE for further investigation of the effect of MP on shotgun assemblies. In SSPACE the key parameter that defines the stringency of the scaffolding is ‘number of links’ (k), i.e. number of independent read pairs that uniquely support a connection between two contigs. We performed SSPACE scaffolding with k equal to 3, 5, 7, 10, 15 and 20.

### Gene content

The protein annotation (v1.2) excluding splice variants of *Brachypodium distachyon* (referred to as Brachypodium) was used as query sequences in a TBLASTN search [30] to assess gene content in contigs and scaffold assemblies. Blast result filtering were carried out as follows: (1) Only query proteins having at least one exon hit with minimum 30 amino acid length and a minimum per cent identity of 70 were considered in the analyses. (2) Duplicated exon hits on one contig/scaffold were removed. Duplicate hits were defined as two or more query hits with identical query start and query end positions, identical mismatches, identical gap length, and identical hit identity. (3) For each query protein, the mean e-value of all hits were calculated and overlapping exon hits (overlapping >5 bp) from proteins with higher e-value were discarded. (4) Two types of gene coverage were calculated: ‘total coverage’ and ‘adjusted coverage’. Total coverage was calculated as the total length of all the hits from a protein query relative to the query sequence length. Adjusted coverage was calculated as the number of unique query amino acid residues with a blast hit in the target sequence(s). To exclude gene hits from repetitive DNA (e.g. TE-associated coding regions) and spurious protein homology, genes with total coverage of >5 and genes with <10% adjusted coverage was not considered in any analyses.

A gene fragmentation index (GFI) was estimated to compare gene space fragmentation in different assemblies. The average blast hit coverage of Brachypodium gene homologs in the entire assembly, referred to as assembly coverage (AC) represents an approximation of the theoretically optimal situation, when each gene is contained within a single DNA sequence (i.e. no fragmentation). The AC estimate was then compared with the average Brachypodium gene coverage per contig or scaffolds, referred to as sequence coverage (SC), to calculate a gene fragmentation index (GFI) defined as (AC-SC)/AC. Hence, the GFI measures gene fragmentation as the difference in percent between SC and AC, and approaches 0 as SC and AC become similar.

### Evaluation of scaffold reliability

As we cannot directly measure the level of scaffolding errors due to the lack of any reference assembly, we estimated the level of scaffold errors by (1) utilizing information from synteny with Brachypodium and (2) comparing the 7BL scaffold assemblies with the sequence content of 50 random BAC clones from 7BL. Because the number of chimeric contigs is assumed to be very low, the level of errors introduced by scaffolding can be estimated by comparing the synteny levels in contigs with synteny in scaffolds of similar sizes. If homologs of two Brachypodium genes are present in a single wheat contig, these homologs have a probability of representing closely linked loci (referred to as neighbouring genes) on the Brachypodium chromosome. This probability depends on the synteny level between wheat and Brachypodium in that exact region. If the scaffolding process does not introduce structural assembly errors, the proportion of neighbouring Brachypodium homologs should be similar in contigs and scaffolds of similar size. In our analyses we defined a neighbour gene pair as genes originating from Brachypodium loci with < =50 genes distance from each other. A bootstrap test was performed to test if the difference in proportions of neighbouring loci in contigs and scaffolds were likely to occur as a consequence of random sampling error. One thousand contig datasets were re-sampled (with replacement) and the *P*-value was calculated as the proportion of bootstrapped contig datasets with equal or lower proportions of neighbour genes as found in scaffolds.

In addition to the synteny approach we also utilized the sequence content of 50 BAC clones originating from 7BL to evaluate scaffold reliability (See Additional file 1 for assembly methods and Additional file 2 for sequence contigs). Raw sequencing reads from 7BL BACs are available upon request. We first identified scaffolds containing sequences derived from the BACs by BLASTN, using a threshold of >99% identity across minimum 2.5 Kb. With the assumption that identified scaffolds truly are derived from one of these 50 BACs, an estimate of scaffold reliability can therefore be defined as the proportion of contigs within a scaffold that originate from a certain BAC. To assess if contigs in scaffolds originate from the BAC we used BLASTN and defined a significant contig-to-BAC hit as having >99% identity across >50% of the contig length. Because longer scaffolds are more likely contain sequences belonging to multiple BACs (i.e. lower proportion of contigs originating from a single BAC) and scaffolding stringency affect scaffold length distribution, we normalized the scaffold reliability by dividing on scaffold length (i.e. proportion of contigs in a scaffold with a BLASTN hit to BAC/scaffold length). Normalized scaffold reliability is hereafter referred to as scaffold reliability index (SRI).

## Results

### Shotgun assembly of 7BS and 7BL

106 and 100 million 100 bp PE reads with an average insert size of 346 bp (7BS) and 362 bp (7BL) (Additional file 3) were generated from the MDA DNA from flow-sorted 7BS and 7BL chromosome arms, respectively (short read archive accession number: ERP002001). Of the mapped read pairs >99.8% were oriented in the assumed FR directions. This represents approximately 59x coverage of 7BS and 37× coverage of 7BL. The assembly with ABySS produced a total of 1,349,563 contigs for 7BS and 4,527,901 contigs for 7BL (Table 1) (contigs are available upon requests). After removing contigs of less than 200 bp, the assemblies were reduced to 178,789 7BS contigs and 328,725 7BL contigs, with an N50 of 2,428 and 1,556 bp, respectively (Table 1). The filtered datasets constituted 13.3% of 7BS and 7.3% of 7BL contigs, representing 57% and 48% of the two chromosome arms assuming a molecular size of 360 Mbp for 7BS and 540 Mbp for 7BL [14], respectively.

**Table 1:** Contig assembly summary statistics

**Arm**	**Contig number**	**N50 (bp)**	**Mean length (bp)**	**Max length (bp)**	**Total (Mbp)**
7BS	1,349,563	842	239	50,938	323
	178,789^*^	2428	1152	50,938	206
7BL	4,527,901	145	144	30,964	652
	328,725^*^	1556	789	30,964	260

^*^Contigs > 200 bp.

### Mate pair data

A total of 445 million 7BS and 478 million 7BL MP read pairs were generated (short read archive accession number: ERP002001), the coverage was estimated to be 88.9x and 63.9× for the short and long arm, respectively (Table 2). Seventy-one per cent of the MPs had both reads mapping to the assembly, 23% of the read pairs only had one read mapping to a contig (i.e. singleton), and about 5% of the MP data did not map to any of the 7B contigs. The between-library variation in the proportions of mapped reads were very low, however the 3 Kb and 5 Kb libraries had slightly smaller proportion of unmapped reads and singletons (2-5% less) compared to the 2 Kb library (data not shown).

**Table 2:** Summary table of mate pair sequence data

**Arm**	**Mate pair library**			**Total pairs**	**Read class**		
	**2 Kb°**	**3 Kb†**	**5 Kb†**		**Pairs**	**Singletons**	**Unaligned**
7BS	2.60*107	2.23*108	1.97*108	4.46*108	71.8%	22.4%	5.9%
7BL	3.13*107	2.32*108	2.16*108	4.79*108	71.2%	23.7%	5.1%

Total numbers of read pairs are given for each MP library. The read pair classification is based on mapping of MP data back to assembled contigs from PE data.

°Roche library, †Illumina library.

**Figure 1:**
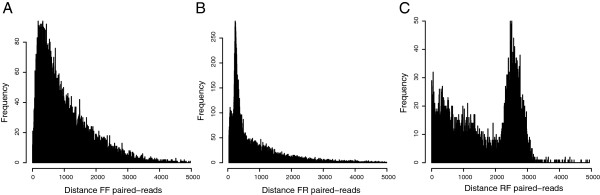
**MP read classes and the estimated insert size distributions for the 3 KB library of 7BS. A**) distribution of insert sizes for the FF reads **B**) distribution of insert sizes for the FR reads **C**) distribution of insert sizes for the RF reads.

MP reads, which map to the same contig, can be classified according to their orientation. In theory, MP should be oriented in a reverse/forward (RF) manner; however, of the MP reads that mapped to one contig, only 15% and 29% were classified as having a RF orientation on 7BS and 7BL, respectively. To better understand the nature of the non-MP oriented read pairs, we estimated insert size based on the mapping information. Figure 1 illustrates the variation and distribution of insert sizes for the RF, FR, and FF/RR oriented MP reads in the 3 Kb library of 7BS (Similar figures for all libraries can be found in Additional files 4 and 5). It is evident that the insert size distribution of the properly oriented MP read pairs represents a mix of the expected size range (a normally distributed peak) in addition to a relatively high proportion of reads with smaller and variable insert sizes. The insert size distribution of the non-MP oriented FF/RR and FR reads does not show the expected normal distribution, but is more similar to a log-normal distribution with a large proportion of reads from short insert size fragments of <1000 bp.

Mapping of MP reads classified as having RF, FR, and FF/RR orientation to the repeat database showed no apparent difference in repeat content (~36% mapped to repeat database, data not shown). It is not uncommon to have PE oriented reads (i.e. FR) in MP libraries (c.f. mate pair library sample preparation guide), but the occurrence of FF/RR reads is more difficult to explain. Hence, we specifically analysed the content of the FF/RR-mapped reads by BLASTN against the NCBI nr database to assess if the FF/RR reads originated from other sources than wheat DNA. All target hits with >80% identity in the NCBI nr nucleotide database were collected and the species information of each hit was extracted. More than 96% of the reads had a best BLAST hit to other grasses (Additional file 6), implying that the FF/RR-mapping reads truly were derived from wheat DNA.

### Effect of MP integration on 7B assemblies

In the process of producing scaffolds, SSPACE enforces stringent criteria for incorporating MP information; MP reads used in SSPACE must have a unique perfect hit in the contig assembly and satisfy the *a priori* defined insert size range. Across all SSPACE assemblies only 1–1.8% of the MP read pairs satisfied the perfect match, read orientation, and insert size criteria (Table 3). Most of the discarded MP reads (63-71%) were classified as having unsatisfied pairing orientation, i.e. either FF/RR or as a pair end read (FR).

**Table 3:** Scaffold assembly summary statistics

**k**	**Arm**	**MP used (%)**	**No. scaffolds**	**Contigs in scaffolds**			**Mean length**	**Sum scaffolds (Mbp)**	Assembly N50* (Kb)
							**(min-max)**		
				**Mean**	**Max**	**%**	**(Kb)**		
3	7BS	0.96	20,654	4.51	38	52	11.2 (1.7-143.1)	168	14.49
	7BL	1.56	31,582	4.33	43	42	9.6 (2.0-117.6)	192	11.15
5	7BS	1.06	17,481	3.81	27	37	10.7 (1.7-129.4)	148	11.03
	7BL	1.41	23,365	3.91	32	28	9.5 (2.2-122.1)	166	8.31
7	7BS	1.14	15,230	3.4	20	29	10.5 (1.72-109.6)	133	9
	7BL	1.48	19,610	3.56	25	21	9.3 (2.3-81.9)	148	6.33
10	7BS	1.24	12,750	3.04	15	22	10.5 (1.8-108.9)	115	7.04
	7BL	1.58	15,896	3.22	20	16	9.3 (2.3-77.7)	128	4.49
15	7BS	1.35	9,733	2,73	12	15	10.7 (2.0-102.4)	92	5.2
	7BL	1.7	12,052	2.89	17	11	9.4 (2.54-67.4)	103	2.84
20	7BS	1.42	7,618	2,55	10	11	10.9 (2.1-73.3)	76	4.2
	7BL	1.79	9,458	2.68	14	8	9.6 (2.8-69.2)	84	1.97

* Including all sequences (contigs + scaffolds).

Even though only 1-2% of MP data was used for scaffolding the assembly N50 was improved substantially at low stringency levels; at k = 3 assembly N50 increased by 6 and 7.2 fold for 7BS and 7BL, respectively (scaffolds are available upon requests). However, as expected, the reduction in assembly fragmentation was dramatically affected when k was increased (Table 3). The number of contigs incorporated in scaffolds decreased from 42% to 8% and from 52% to 11% for 7BL and 7BS, respectively, and the total number of scaffolds decreased by ~70% when increasing k from 3 to 20. Furthermore the total number of residues included in scaffolds was reduced from ~40% to 18% of the total chromosome length (Table 3). The mean scaffold length however, was not affected much when k was increased due to a change in the distribution of length of contigs included in scaffolds; as k increased, the proportion of long contigs included in scaffolds also increased. Scaffold content was strongly biased towards gene containing contigs. Although a maximum of 40-50% of 7BS and 7BL contigs were incorporated into scaffolds (Table 3), as many as 75% (k = 20) and 95% (k = 3) of sequences containing full length genes (> = 70% Brachypodium homolog coverage) were included in scaffolds.

**Figure 2:**
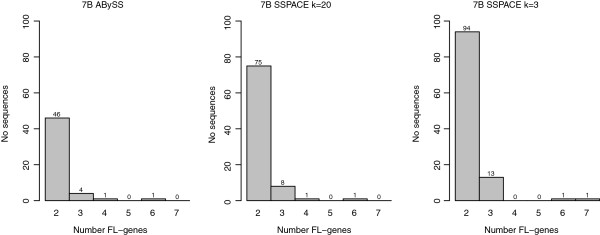
MP effect on sequences containing multiple full length (FL) genes.

Next we assessed how MP data helped to join fragmented gene parts into more complete gene sequences by calculation of a gene fragmentation index (GFI) and counting full length genes contained in single sequences. After removing all BLAST hits with <10% coverage of a Brachypodium protein the AC and GFI were 0.54/0.17 and 0.49/0.21 in the 7BS and 7BL contig assemblies, respectively, while the scaffold assembly GFI ranged between 0.09-0.14 (Table 4). The MP integration also increased the number of full length genes in the range of 10–16% and 20-30%, depending on how a full length gene was defined (Table 4). In addition to aiding the joining of exons from fragmented gene sequences, MP information also helps to link genes belonging to different contigs together in multigene containing scaffolds (containing > =2 full length genes), and thereby helps ordering genes relative to each other. A modest effect on gene linking was observed after the MP integration in the 7BS and 7BL assemblies (Figure 2). The number of sequences containing 2 and 3 genes increased by 2–3 fold when applying k = 3 and by 1.5-2 fold when applying k = 20, compared to the contig assembly. However, virtually no changes was observed for sequences containing >3 genes. Moreover, the gene composition in scaffolds containing multiple genes were not random with respect to the length of the gene, but showed a clear bias towards shorter genes. For example, for the SSPACE k = 5 assembly the mean gene length in scaffolds and contigs with 3 or more genes were much shorter (contigs 462 bp/scaffolds 722 bp) than the mean CDS in sequences with 2 genes (contigs = 1,354 bp/scaffolds = 1,604 bp).

**Table 4:** Gene content in ABySS and SSPACE assemblies

**Assembly**	**Arm**	**Brachypodium homologs**	**Brachypodium homolog coverage**^**1 **^**(mean)**	**GFI**^**2**^	**Full length genes**	
					Coverage*	**Start-stop†**
		**(>30 aa, >70 pident)**				
SSPACE k3	7BS	1029	0.49	0.09	449	193
	7BL	1539	0.44	0.10	551	224
SSPACE k5	7BS	1038	0.49	0.09	445	193
	7BL	1545	0.43	0.12	547	227
SSPACE k7	7BS	1032	0.49	0.09	449	196
	7BL	1551	0.43	0.12	535	221
SSPACE k10	7BS	1038	0.49	0.09	447	195
	7BL	1555	0.43	0.12	533	215
SSPACE k15	7BS	1040	0.48	0.12	436	186
	7BL	1576	0.42	0.14	529	217
SSPACE k20	7BS	1048	0.47	0.13	433	183
	7BL	1574	0.42	0.14	516	205
Contigs	7BS	1071	0.45	0.17	403	160
	7BL	1621	0.39	0.21	457	162

^1^Mean coverage per sequence (contig/scaffold) of Brachypodium homologs based on blast analyses (see methods).

^2^ Gene Fragmentation Index (GFI) is defined in the methods section.

* TBLASTN hits covering > =70% of a homologous Brachypodium protein in a single contig/scaffold.

† TBLASTN hits covering an entire Brachypodium protein (+ − 10aa) in a single contig/scaffold.

### Scaffold content reliability

**Figure 3:**
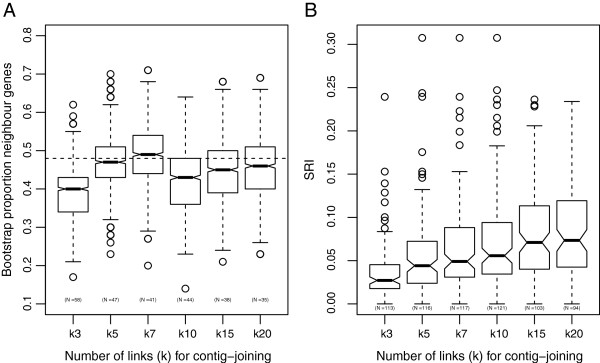
**Evaluation of scaffold reliability. A**) Scaffold reliability evaluated by the proportion of conserved syntenic relationship to the Brachypodium genome. **B**) Scaffold reliability evaluated by comparisons of sequence content in BACs and scaffolds. SRI = scaffold reliability index (defined in method section).

Integration of mate pair data can lead to misassemblies due to erroneous coupling of contigs. We took advantage of the Brachypodium model genome to estimate the scaffolding error levels based on synteny. Scaffolds and contigs containing 2 full length Brachypodium homologs were identified and the proportion of neighbour genes based on the location in the Brachypodium genome was calculated. The frequency of neighbouring genes in contigs were 0.48 and in scaffolds between 0.4 (k = 3) and 0.49 (k = 7) (Figure 3A). Furthermore, the bootstrap tests did not reject the null hypotheses that contigs have higher proportion of neighbouring genes at α = 0.05, even for the scaffolds produced at the lowest stringency (k = 3, *P* = 0.15). Taken together, our synteny error rate estimates do not indicate high rates of random contig joining at any level of SSPACE stringency. Scaffold reliability estimation based on sequence content in BAC clones reflected a slightly different and more pronounced effect of changing scaffolding stringency. The median scaffold reliability index increased progressively from the k = 3 (0.38) to k = 20 (0.86) assemblies (Figure 3B), indicating a higher scaffold correctness in SSPACE assemblies with high k-values.

### Effect of MP coverage

**Figure 4:**
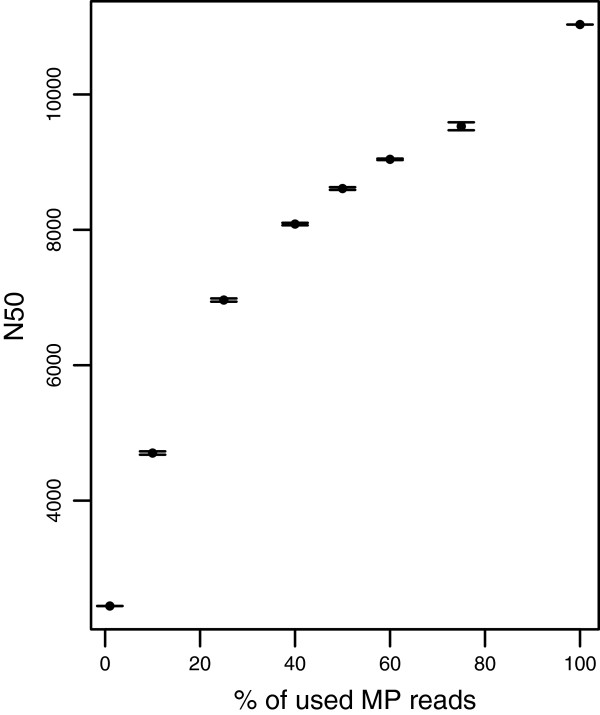
**Effect of MP sequencing depth on assembly N50.** Only 7BS was used to estimate the MP coverage effect.

Increasing sequence coverage of MP data has impact on assembly statistics, but upon reaching certain coverage, added value of additional MP sequencing may not justify the cost of data generation. It is therefore important to evaluate the effect of MP coverage on our assembly metrics. To assess the relationship between MP coverage and assembly improvement, we generated randomly reduced datasets of our 7BS MP libraries with 1, 10, 25, 40, 50, 60 and 75% of original MP coverage and generated scaffolds with the number of links parameter k = 5. Three random sub-sets of MP data were generated for each reduced level of coverage. Interestingly, little change was observed in assembly statistics until the coverage was reduced with 50% (Figure 4), and a corresponding 22% reduction in N50 was observed. Even less effect of decreasing MP coverage was seen in statistics for gene content information in reduced MP coverage assemblies. For example, reducing the MP coverage by 90% only produced a 25% reduction in the number of full length genes (427) while the 50% reduced coverage assembly contained 7% fewer full length genes (435).

## Discussion

### Modest effect of MP integration in 7B shotgun assemblies

*De novo* assembly of shotgun sequences from large plant genomes like wheat remains a challenging task, mainly due to prevalence of repetitive DNA [31]. Its presence can lead to complex, misassembled rearrangements and the collapse of reads coming from distinct copies of repetitive DNA into single assembled sequences [23] which results in contracted and fragmented assemblies. Ideally, genomes with high repeat content should therefore be assembled using reads longer then the length of the repeats. Wheat consists of >80% TE-derived repetitive DNA with a mean length of ~4600 bp [24], far longer than the read length of sequence reads from next generation sequencing data. One assembly strategy to reduce the fragmentation generated by repetitive DNA is therefore to link neighboring chromosome regions belonging to different sequence contigs with long insert size MP reads that bridge the repetitive segments.

Our results show that integration of MP data in shotgun assemblies of flow-sorted wheat chromosomes improves assembly contiguity and decrease gene space fragmentation, but that the degree of assembly statistics improvement is greatly affected by scaffolding stringency (Table 1, Table 3). For example, at low stringency the assembly N50 increased by 6–7.5-fold, while at the highest stringency (k = 20) N50 was only increased by 1.3-1.8 compared to contig assemblies. Although a negative correlation between stringency and assembly improvements also was observed for the gene space statistics, the MP effect on gene space were less affected by scaffold assembly stringency compared to the N50 statistic (Table 4). This is likely explained by the fact that genes are more often found in longer contigs, hence even at high stringency a large proportion of the gene containing contigs were joined into scaffolds.

Even for the least stringent scaffold assembly, the assembly improvement for 7B does not seem to be in the same magnitude as reported for some recently sequenced plant genomes. For the cucumber (1C = 367 Mbp) and cacao (1C = 430 Mbp) genomes, addition of long insert libraries improved N50 by 14-fold (172 Kb) and 60-fold (473.8 Kb), respectively [4,9]. Assembly metrics from these shotgun assemblies is difficult to compare directly due to the use of different sequencing platforms, different PE and MP libraries, and different sequencing coverage. However, it is possible to estimate the MP-effectiveness based on the total gain in N50 per Kb of MP insert size length (N50 gain/max MP insert size). For cucumber and cacao these numbers are 14/2 Kb and 60/8 Kb compared to for example 5/5 Kb in wheat k = 5.

Potato is another recently published plant genome [25] for which different MP libraries were added to improve assembly. Integration of a 2 Kb MP library produced a 3-fold increase in N50, while using both 2 Kb + 5Kb MP libraries increased the N50 by 8-fold. Comparable metrics were obtained in 7B SSPACE assemblies using k = 5; the 2 Kb MP libraries produced 1.9- and 2.6-fold changes in N50 for 7BS and 7BL, respectively, while the final N50 fold change was ~5 after addition of the 5 Kb MP data. Thus, even though the actual scaffold N50 was much higher in potato after adding 2 + 5 Kb MP libraries (173 Kb) compared to the 7B assemblies with 2 + 3 + 5 Kb MP data (7BS = 11 Kb/7BL = 8.3 Kb), the relative N50 gain was not that different.

The modest impact of MP data in the chromosome 7B assemblies compared to other plant genomes could be explained by the inherent repeat characteristics of the wheat genome. While the wheat genome consists of >80% TE-derived repetitive DNA [24], potato, cacao and cucumber genomes are much smaller and all have <35% TE-derived repetitive DNA [32]. This difference will undoubtedly cause large differences in the effect of MP data on assembly contiguity. Another reason for the relatively low impact of MP data on the scaffold N50 could be related to the quality of the MP data. Only a small fraction (~1%) of the MP reads from MDA chromosomal DNA satisfied requirements of SSPACE for being included in scaffold construction, mostly due to a very high portion of reads having a different orientation or discrepancy between expected and estimated insert size for MP reads (Table 2). Lastly, the fragmentation level of the contig assembly is important for the MP effect. It is evident that small contigs have less chance of being put into scaffolds due to the fact that small sequences will have few MP reads originating from them. An improved contig assembly N50, for example by increasing PE sequencing coverage or adding additional PE libraries with different insert sizes, could therefore be a good strategy to be able to include a larger proportion of the contig assembly into scaffolds, and hence increase scaffold N50.

### Scaffold reliability and assembly stringency

Assembly errors can be introduced at the scaffolding stage when the software has to choose between two similar solutions and falsely connects contigs from non-adjacent chromosome regions or links two adjacent contigs in the wrong orientation. Our synteny- and BAC-based scaffold reliability estimates provides measures of reliability at two types of different genomic landscapes. Our synteny approach did not detect signatures of erroneous contig joining in small scaffolds from gene dense regions in the assemblies; however when using sequence contents from 50 BACs to assess scaffold reliability a strong correlation between estimated scaffold reliability and scaffold assembly stringency was observed (Figure 3). We interpret these differences in test conclusions to reflect that scaffolds from non-genic genomic regions are more prone to contain errors (especially at low stringency parameters), likely due to higher content of repetitive DNA in the intragenic space.

### The origin of erroneous MP orientation

The MP data contained a high percentage of forward-reverse reads (i.e. PE) as well as contamination of read pairs that map in the same direction (FF/RR) (Table 2). The high proportion of FR reads in our MP data is most likely explained by contamination with PE reads, which represent non-biotinylated fragments that were not removed during the wash step in library preparation (c.f. mate pair library sample preparation guide). This is supported by the fact that these PE oriented reads have a smaller estimated insert size of around 500 bp (Figure 1, Additional files 4 and 5). The origin of MP reads oriented in FF/RR direction, which make up ~38% of the total MP data, is less obvious. There is no evidence for FF/RR reads containing non-wheat DNA contamination, nor do the FF/RR reads have increased proportions of reads from TE-repetitive DNA. Moreover, since the 2 Kb and 3/5 Kb libraries were produced and sequenced by different labs using different protocols it is highly unlikely that systematic technical errors have been introduced. One possible explanation to the high FF/RR fraction is that they originate from rearranged DNA generated in the multiple displacement amplification (MDA) step, which was used to increase DNA amount after chromosome flow-sorting. It has been shown that MDA generates genomic rearrangement in the amplified DNA with a frequency of 1 rearrangement per 10 Kb, and majority of chimeras are inverted sequences [18]. In a *de-novo* assembly of a single bacteria cell MDA, >50% of the MP pairs were chimeric pairs [33]. Hence, even though MDA has proven to be very useful to prepare DNA from flow sorted chromosomes for single-end and short insert size PE sequencing [20,21,34], the use of MDA DNA in long insert size MP library construction and scaffolding might not be an optimal strategy for wheat genome scaffolding. Another limitation due to the high proportion of FF/RR pairs is that it restricted us from using any type of scaffold-assembler. For example, when trying to integrate MP data using ABySS, the software did not handle the large proportion of the MP reads with non-MP orientation.

## Conclusion

The wheat chromosome 7B was sequenced and assembled using PE reads with an insert size of ~350 bp in combination with 2, 3 and 5 Kb MP libraries. MP integration improved both assembly contiguity and reduced fragmentation of the gene space, but only to a modest extent. Scaffold reliability increased with increasing assembly stringency, emphasizing the need to use high stringency scaffolding parameters to avoid scaffolding errors. Scaffold assemblies of 7BS with reduced MP coverage showed that MP sequence coverage of ~40-50× would be sufficient to produce assemblies with slightly reduced N50 but comparable results for gene space improvement compared to the full coverage assembly (89×). In conclusion, MP assembly improvements was lower than for other recently assembled plant genomes, possibly due to the extreme repeat content of wheat, high fragmentation of contig assemblies, and the use of MDA DNA to construct MP libraries.

## Competing interest

The authors declare that they have no competing interests.

## Authors’ contributions

TB carried out bioinformatics on scaffolding and participated in writing the manuscript. BZ estimated insert sizes of MP libraries, participated in data analyses and helped draft the manuscript. JW and MC performed assembly of PE reads, and participated in drafting the manuscript. TA, CB and FP coordinated and carried out the MP sequencing and helped to draft the manuscript. HS- carried out isolation and preparation of MDA DNA, and helped draft the manuscript. JD led the work on flow-sorting of 7B chromosome, and was involved in drafting the manuscript. MK was responsible for the sequencing of one MP library, carried out data analyses and helped draft the manuscript. SL and OAO helped coordinate the study, participated in data analyses, and helped draft the manuscript. SRS carried out bioinformatics analyses of gene content, helped to draft the manuscript, and was responsible for the final version of the manuscript. All authors read and approved the final manuscript.

## Supplementary Material

Additional file 1 7BL_BAC_assemblies. Click here for file

Additional file 2 BAC_contigs_from_50_BACs. Click here for file

Additional file 3 PE_insert_size_distributions. Click here for file

Additional file 4 7BS_libraries. Click here for file

Additional file 5 7BL_libraries. Click here for file

Additional file 6 Distribution_of_ff_reads_genus_hits. Click here for file
