# Supplementary material for: Integration of mate pair sequences to improve shotgun assemblies of flow-sorted chromosome arms of hexaploid wheat
Source: BMC Genomics. 2013 Apr 4;14:222. doi: 10.1186/1471-2164-14-222 (PMC3622640; doi:10.1186/1471-2164-14-222)
Supplement: Additional file 1 — 7BL_BAC_assemblies. [file 1471-2164-14-222-S1.pdf]

Assembly of 7BL BAC clones

DNA from 50 BACs belonging to 7BL (`TaaCsp7BLhA`, http://olomouc.ueb.cas.cz/dna-libraries/cereals) was isolated and sequenced on Illumina HiSeq2000. BAC-specific paired-end reads with 500bp insert size were cleaned by removing low quality, contamination and PCR duplications reads. Assembly and paired-end read scaffolding was carried out independently for each BAC clone with SOAPdenovo v1.06. Assemblies of BAC were optimized by testing various *k-mer* sizes (33-63) and selecting the assembly with the largest N50.
