# Supplementary figures and images for: Integration of mate pair sequences to improve shotgun assemblies of flow-sorted chromosome arms of hexaploid wheat
Source: BMC Genomics. 2013 Apr 4;14:222. doi: 10.1186/1471-2164-14-222 (PMC3622640; doi:10.1186/1471-2164-14-222)

7BS PE insert size distribution

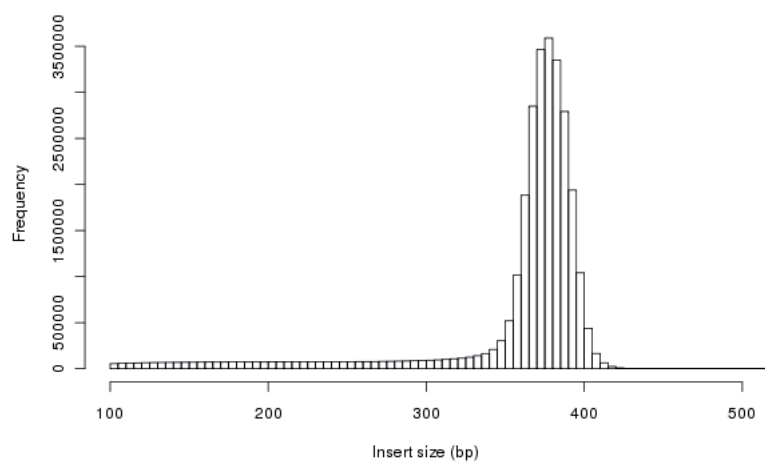

7BL PE insert size distribution

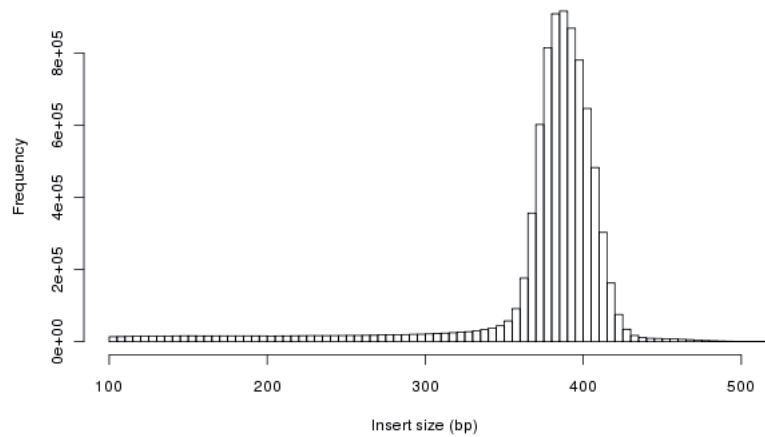

Supplement: Additional file 3 — PE_insert_size_distributions. [file 1471-2164-14-222-S3.pdf]

The estimated insert size distributions for 7BS libraries

2kb

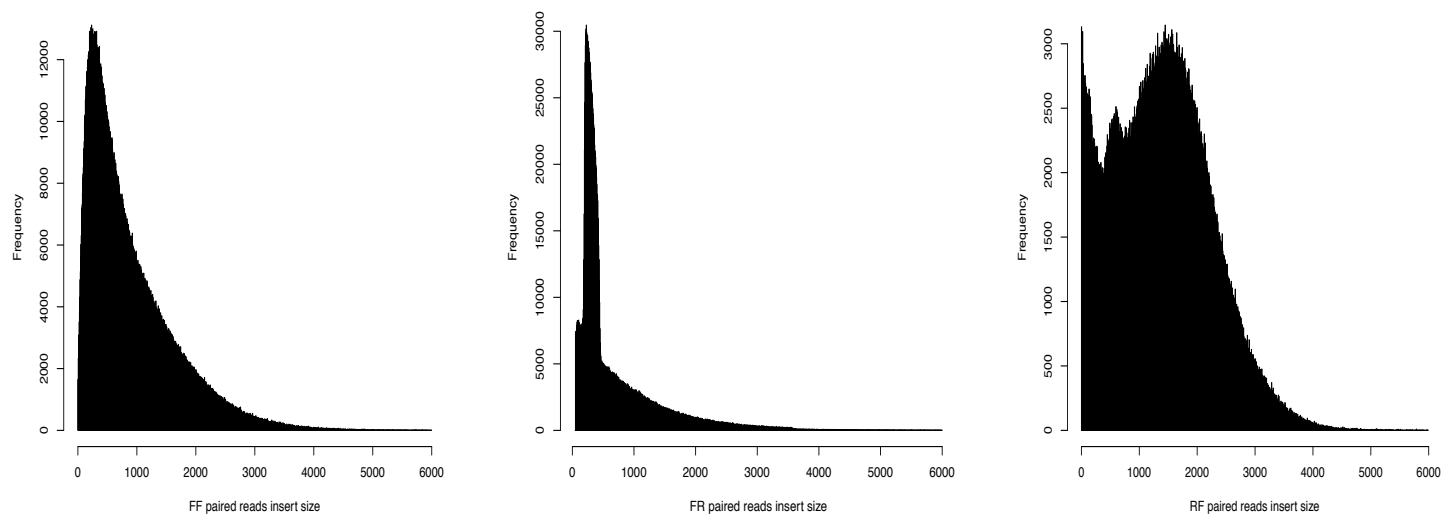

3kb

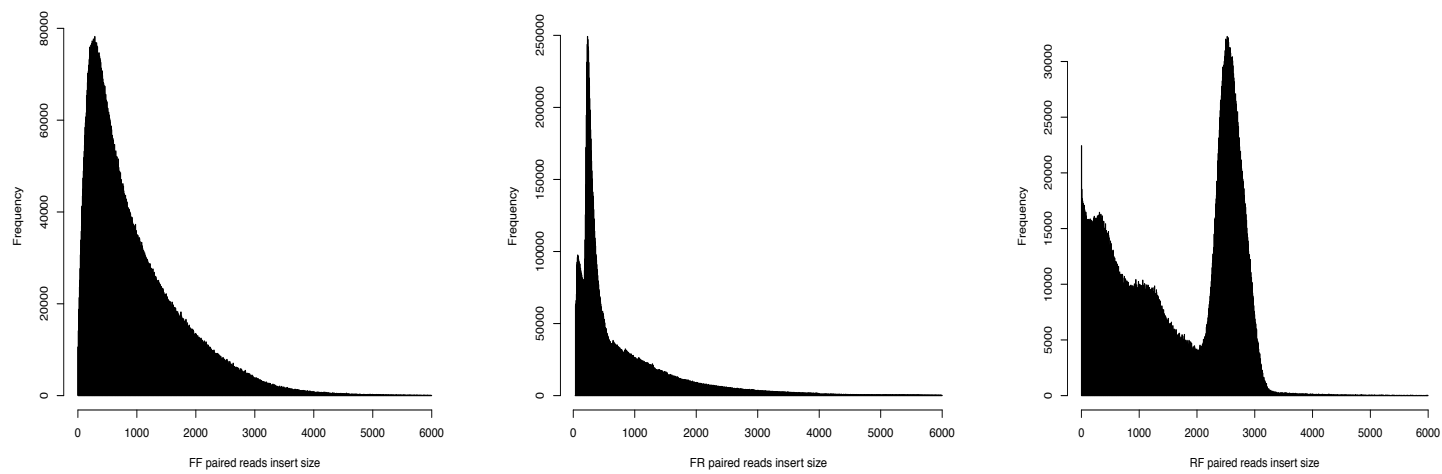

5kb

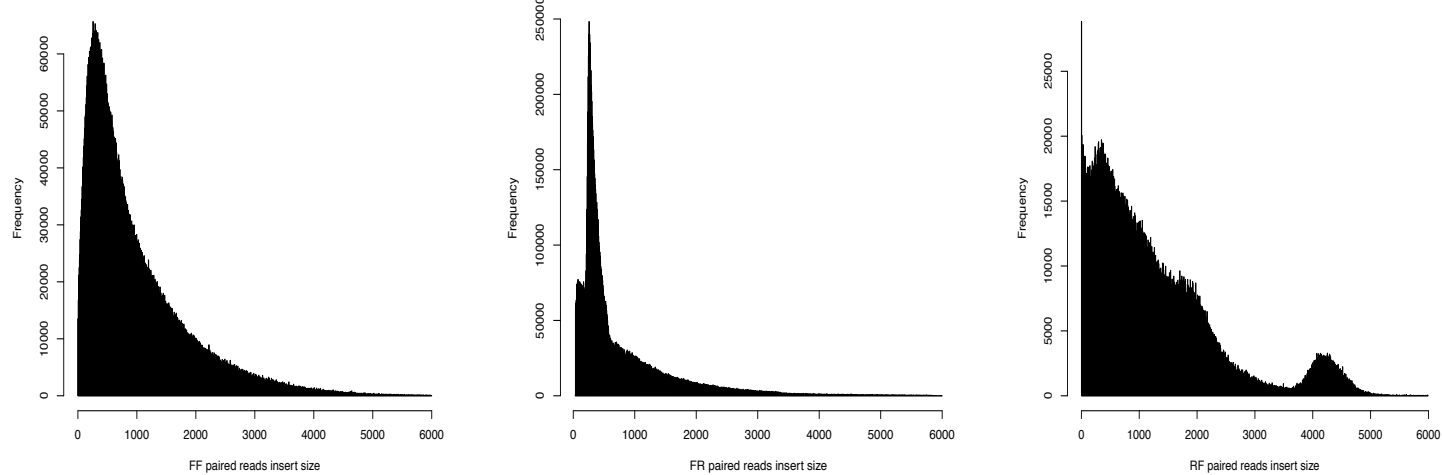

Supplement: Additional file 4 — 7BS_libraries. [file 1471-2164-14-222-S4.pdf]

## The estimated insert size distributions for 7BL libraries

2kb

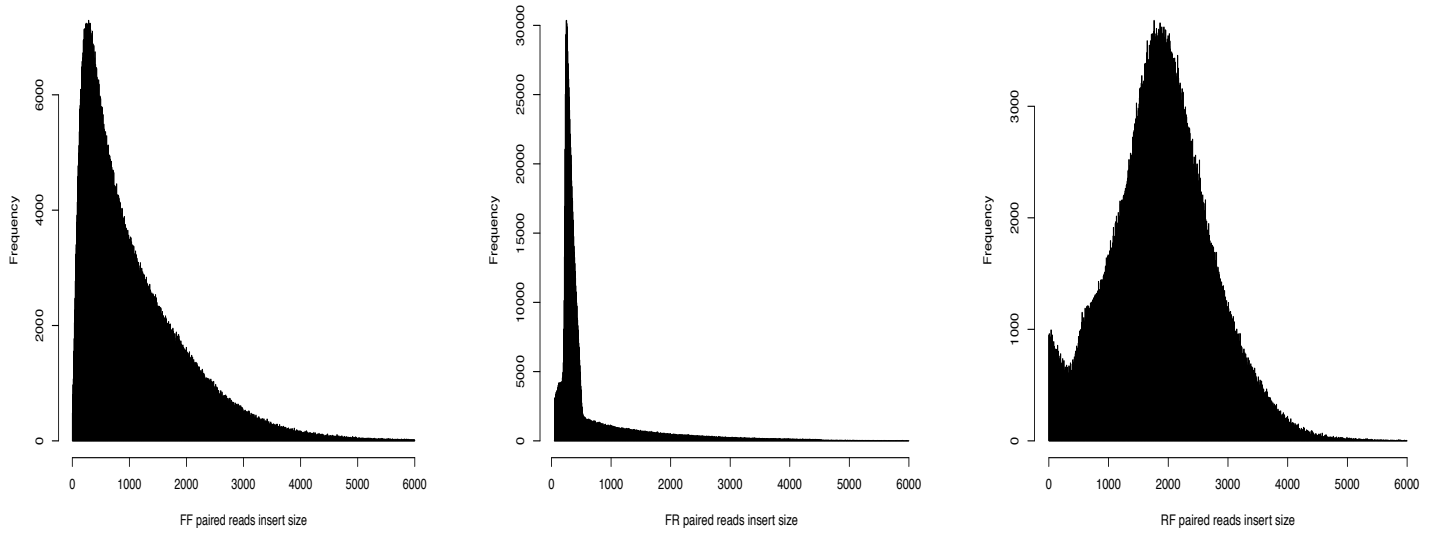

3kb

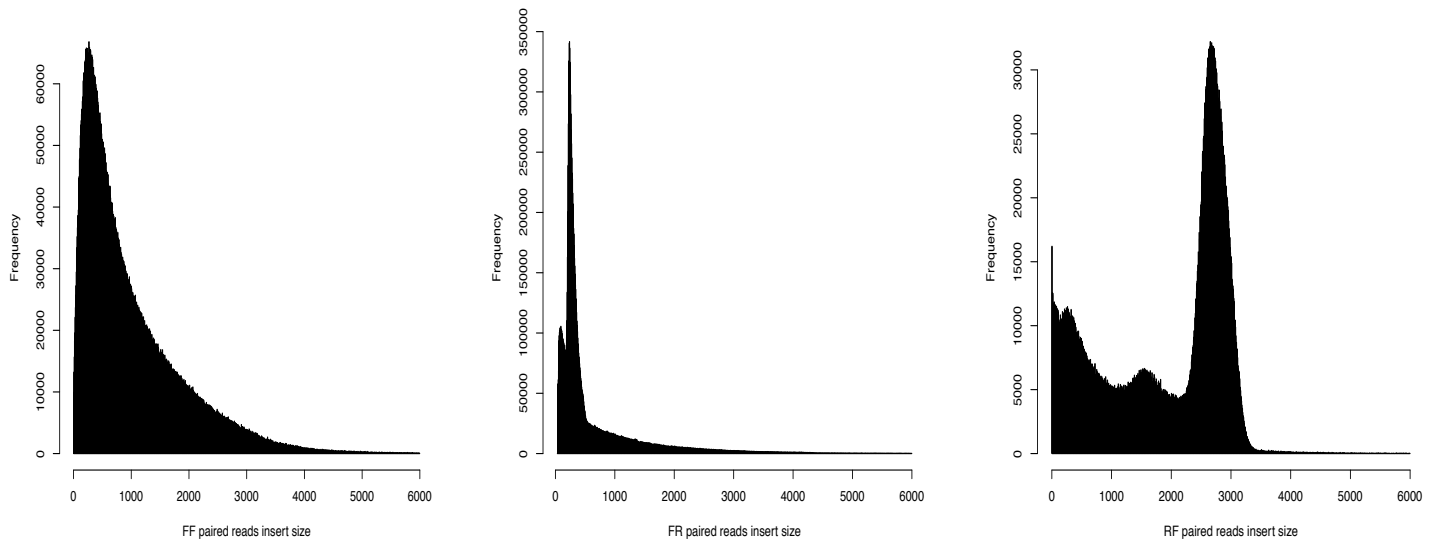

5kb

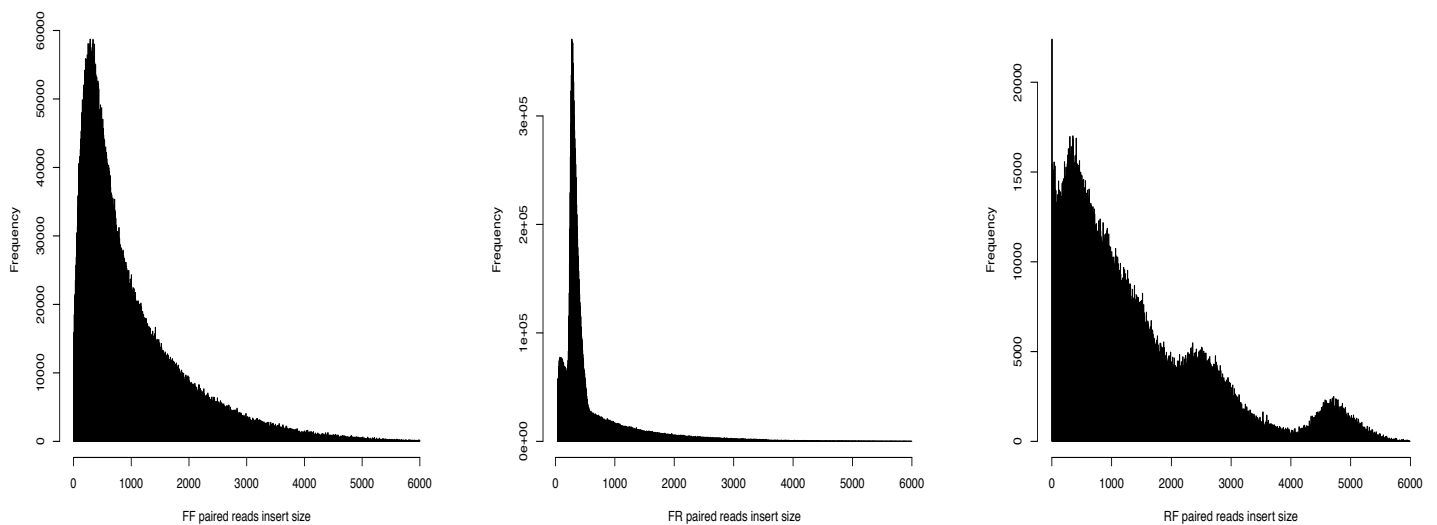

Supplement: Additional file 5 — 7BL_libraries. [file 1471-2164-14-222-S5.pdf]

## FF-reads blastn hits

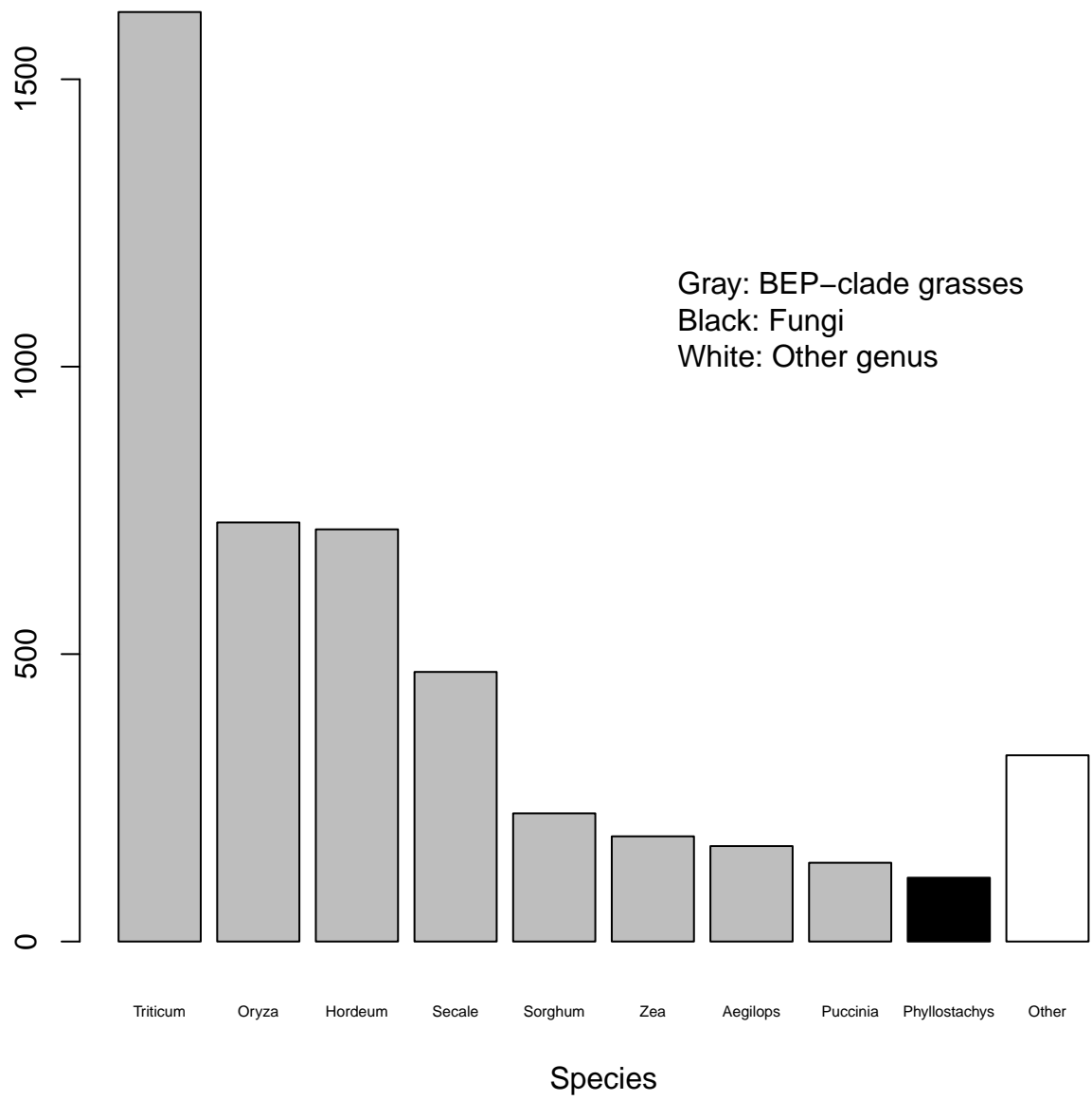

Supplement: Additional file 6 — Distribution_of_ff_reads_genus_hits. [file 1471-2164-14-222-S6.pdf]
